# HIV triggers immunoregulatory dendritic cells and regulatory T cells through the non-canonical NF-kB pathway

**DOI:** 10.1186/1742-4690-9-S2-P184

**Published:** 2012-09-13

**Authors:** O Manches, MV Fernandez, J Plumas, L Chaperot, N Bhardwaj

**Affiliations:** 1 NYU Cancer Institute, New York City, NY, USA; 2 Inserm, U823, Immunobiologie et Immunotherapie des Cancers, La Tronche, France

## Background

HIV stimulates plasmacytoid dendritic cell (pDC) through TLR7 and induce the secretion of high levels of IFNα. pDC stimulated by HIV also upregulate the expression of the enzyme indoleamine 2,3 dioxygenase (IDO). IDO is critical for the induction of regulatory T cells (Treg) by HIV-activated pDC. We investigated the molecular mechanisms of IDO induction and its consequences for Treg function.

## Methods

The cells used were purified primary pDC and the GEN pDC cell line. A combination of siRNA knock-down, immunoprecipitation of TLR signaling pathway molecules, IDO promoter engineering and chromatin immunoprecipitation was used to determine the molecular mechanisms of IDO induction in pDC. To analyze Treg function and interaction with conventional DC (cDC), blocking antibodies to CTLA- 4 and CTLA-4-Ig were used.

## Results

We demonstrate that HIV induces activation of the non-canonical NF-kB pathway in pDC, and is essential for IDO induction. TLR7 triggering induces recruitment of TRAF3 to the TLR-MyD88 complex, followed by release of NIK and phosphorylation of IKKα. Activation of the non-canonical NF-κB pathway culminates in p52/RelB nuclear translocation and binding to the IDO promoter.

Furthermore, IDO-expressing pDC trigger the generation of Treg, which dampen cDC activation through CTLA-4. CTLA-4 also induces IDO expression in cDC in a NIK-dependent fashion, allowing cDC to induce Treg from naïve CD4+ T cells.

## Conclusion

The non-canonical NF-kB pathway plays a central role in regulating IDO expression in pDC and cDC upon HIV infection, and may be a potential target for regulating Treg activity in chronic or acute HIV infection.

